# Intrathecal pemetrexed chemotherapy combined with systemic therapy in patients with non-small cell lung cancer and leptomeningeal metastases: a retrospective study

**DOI:** 10.3389/fonc.2025.1545174

**Published:** 2025-04-07

**Authors:** Wenjuan Zhong, Longqiu Wu, Zhengang Qiu, Wei Yu, Linfang Liu, Huaqiu Shi, Shugui Wu

**Affiliations:** ^1^ Department of Oncology, The First Affiliated Hospital of Gannan Medical University, Ganzhou, Jiangxi, China; ^2^ Jiangxi Clinical Medical Center for Cancer, Ganzhou, Jiangxi, China; ^3^ The First Clinical Medical College, Gannan Medical University, Ganzhou, Jiangxi, China; ^4^ Department of Oncology, The Affiliated Ganzhou Hospital, Jiangxi Medical College, Nanchang University, Ganzhou, Jiangxi, China

**Keywords:** non-small cell lung cancer, leptomeningeal metastases, pemetrexed, intrathecal chemotherapy, EGFR

## Abstract

**Background:**

Leptomeningeal metastases (LM) in non-small cell lung cancer (NSCLC) present a challenging prognosis, with systemic therapies often limited by the blood-brain barrier. However, intrathecal pemetrexed injections can increase intracranial drug concentrations, aiding in disease control.

**Objective:**

To evaluate the efficacy and safety of combining intrathecal pemetrexed with systemic therapy in patients with NSCLC and LM.

**Methods:**

Thirty-one patients with NSCLC and LM who received intrathecal pemetrexed chemotherapy between 2018 and 2022 at First Affiliated Hospital of Gannan Medical College were retrospectively reviewed.

**Results:**

Of the 31 patients enrolled, six had LM at initial diagnosis. The median number of intrathecal pemetrexed injections was 4 (2-26), with an intracranial control rate of 87.1% (27/31). Median iPFS was 9 months (95% CI: 2.77-15.23), and median iOS was 12 months (95% CI: 5.94-18.06 months). Most adverse events (AEs) were grade 1-2, with four (12.9%) grade 3 AEs (including two cases of grade 3 leukopenia; one, grade 3 diarrhea; one, grade 3 interstitial pneumonitis). Univariate and multivariate analyses showed that the combination of bevacizumab (p<0.05) and an Eastern Cooperative Oncology Group(ECOG) score of ≤ 1 (p<0.05) were favorable prognostic factors for survival.

**Conclusion:**

Intrathecal pemetrexed injections combined with systemic treatment demonstrated significant therapeutic efficacy and manageable safety in NSCLC patients with LM.

## 1 Introduction

Leptomeningeal metastases (LM) occur when tumor cells spread into the subarachnoid space and soft meninges through the bloodstream, direct seeding, or via cranial nerves and spinal nerves (1). The incidence of LM in patients with advanced non-small cell lung cancer (NSCLC) is 3% to 5%, with lung adenocarcinoma accounting for 84% to 96% (2, 3) of cases. Additionally, patients with epidermal growth factor receptor (EGFR) mutations (EGFRm) are more likely to develop LM (4, 5). Once LM occur, the prognosis is extremely poor, with a median survival of only 3-6.6 months (6, 7). Currently, there is no standardized treatment protocol for LM, and the available therapeutic approaches include radiotherapy, targeted therapy, chemotherapy, and immunotherapy; however, the efficacy of one treatment alone remains unsatisfactory. Targeted agents, particularly third-generation epidermal growth factor receptor tyrosine kinase inhibitors (EGFR-TKIs), have a high blood-brain barrier (BBB) penetration rate and show significant efficacy in the treatment of patients with EGFRm NSCLC (8, 9). However, acquired resistance may develop over time. Intrathecal chemotherapy bypasses the BBB and enables direct delivery of chemotherapeutic agents to the subarachnoid space, providing a highly targeted and effective treatment approach. Conventional drugs for intrathecal injection include methotrexate and cytarabine, however, their therapeutic efficacy remains unsatisfactory.

Pemetrexed is an antimetabolic anticancer drug that can block the cell cycle in the S phase, effectively inhibiting the growth of tumor cells. It is a first-line chemotherapeutic agent for patients with advanced lung adenocarcinoma (10). Patients with EGFRm NSCLC who developed LM experienced longer survival when treated with pemetrexed than those who did not receive pemetrexed (13.7 months vs 4.0 months) (11). A low dose of pemetrexed has been shown to achieve therapeutically high and sustained cerebrospinal fluid (CSF) concentrations in a rat model of intrathecal injection (12). A phase I clinical trial of intrathecal pemetrexed chemotherapy as a salvage treatment of patients with NSCLC and LM showed a clinical response rate of 31% (4/13) and a disease control rate of 54% (7/13) with a dosage of 10 mg (13). Results of another clinical trial demonstrated that intrathecal pemetrexed chemotherapy had a clinical efficacy of 84.6% (22/26), with two patients achieving complete remission and seven patients achieving partial remission (median OS, 9.0 months) (14). These studies suggest that intrathecal pemetrexed has good efficacy in patients with NSCLC and LM; however, limited reports on intrathecal pemetrexed chemotherapy exist. Therefore, we conducted a retrospective study to evaluate the efficacy and safety of intrathecal pemetrexed chemotherapy combined with systemic therapy in patients with NSCLC and LM.

## 2 Materials and methods

### 2.1 Patients

The present study included 31 patients diagnosed with NSCLC and LM who were admitted to the First Affiliated Hospital of Gannan Medical College between January 1, 2018, and December 31, 2022. Inclusion criteria included the following: (i) patients with pathologically confirmed NSCLC; (ii) patients underwent CSF puncture examination and enhanced head magnetic resonance imaging (MRI); (iii) patients were diagnosed with LM according to the European Society for Medical Oncology-European Association of Neuro-Oncology guidelines and received at least two doses of intrathecal pemetrexed chemotherapy. Exclusion criteria included the following: (i) patients who discontinued treatment; (ii) patients with more than two primary tumors; (iii) patients with missing follow-up information. Intracranial progression-free survival (iPFS) was defined as the time from LM diagnosis to tumor progression, while intracranial overall survival (iOS) was defined as the time from LM diagnosis to either death or the last follow-up. This study was approved by the Ethics Committee of the First Affiliated Hospital of Gannan Medical College.

### 2.2 Data collection

Patients’ clinical data were collected from the electronic medical record database, including information such as age, sex, smoking, Eastern Cooperative Oncology Group (ECOG) score, histological type, TNM stage, gene mutation status, brain-enhanced MRI, CSF cytology, treatments before and after LM diagnosis, and adverse drug reactions after pemetrexed injection. Univariate and multivariate analyses were performed in patients using Cox regression models to clarify prognostic correlates.

### 2.3 Intrathecal chemotherapy

After the onset of LM, all patients were treated with intrathecal pemetrexed injections in combination with systemic therapy. Pemetrexed was administered uniformly through lumbar puncture at a dose of 20-30 mg per dose. The frequency of intrathecal injections was 1-2 times in the first week, 2-4 times in the first month, and 1-2 times every month thereafter. Intrathecal injection therapy could only be discontinued if CSF cytology was negative for more than 3 consecutive tests, if adverse drug reactions became intolerable, if patients refused to continue therapy, or if the disease progressed. Before pemetrexed, dexamethasone (5 mg) was injected intrathecally. All patients should be supplemented with folic acid and vitamin B12.

### 2.4 Evaluation of treatment response and adverse events

We comprehensively assessed the patient’s treatment response using intracranial neurological symptoms, cranial enhancement MRI, CSF cytology, and Karnofsky Physical Status Score (KPS) according to the Response Assessment in Neuro-Oncology (RANO) - LM radiological criteria (15). Imaging assessments were performed independently by two experienced radiologists and AEs were graded using the National Cancer Institute Common Terminology Criteria for Adverse Events (version 4.0).

### 2.5 Follow up

Patients were followed-up via telephone or electronic case system, and those who could not be contacted were considered lost to follow-up.

### 2.6 Statistical methods

Statistical analyses were performed using SPSS version 24.0. Categorical variables were analyzed using either the Pearson χ^2^ test or the Fisher exact test. Survival was calculated using the Kaplan–Meier method with a 95% confidence interval. The Cox proportional hazards regression model was employed to perform univariate and multivariate prognostic analyses on patients’ sex, age, smoking status, MRI, Gene mutation, ECOG score, combined metastases, CSF pressure, CSF protein levels, radiotherapy, and combination with bevacizumab therapy, and P<0.05 was considered statistically significant.

## 3 Results

### 3.1 Baseline characteristics of the patients

All 31 patients had lung adenocarcinoma, including 15 males and 16 females, aged 42-75 years, with a median age of 58.3 years. Among them, 14 were smokers, and 17 were non-smokers. The gene mutation status was EGFR 21 L858R mutation in 14 cases, EGFR 19 Del in seven cases, EGFR20 ins in four cases, EGFR T790M in one case, negative driver gene in three cases, KRAS mutation in one case, and ROS1 fusion in one case. At the time of diagnosis of LM, 23 cases (74.19%) had an ECOG score of 0-1, eight (25.81%) had an ECOG score of ≥2, 18 cases (58.06%) had brain metastases, and 21 cases (67.74%) had extracranial metastases (**Table 1**).

**Table 1:** Basic characteristics of patients with NSCLC and leptomeningeal metastases (N=31).

Factor	Number of patients (%)
Age	
<60	16 (51.61%)
≥60	15 (48.39%)
Sex	
Male	16 (51.61%)
Female	15 (48.39%)
ECOG score	
0-1	23 (74.19%)
≥2	8 (25.81%)
Smoking	
Yes	14 (45.16%)
No	17 (54.84%)
MRI	
Negative	5 (16.13%)
Positive	26 (83.87%)
Brain metastases	
Yes	18 (58.06%)
No	13 (41.94%)
Extracerebral metastasis	
Yes	21 (67.74%)
No	10 (32.26%)
Gene mutation	
EGFR21 L858R	14 (45.17%)
EGFR19DEL	7 (22.58%)
EGFR T790M	1 (3.22%)
EGFR 20ins	4 (12.90%)
ROS1	1 (3.22%)
KRAS	1 (3.22%)
Negative	3 (9.68%)
High protein in CSF	
Yes	21 (67.74%)
No	10 (32.26%)
CSF pressure	
High	13 (41.94%)
Normal	18 (58.06%)
Combined treatment after LM	
Targeted therapy	24 (77.42%)
Chemotherapy	7 (22.58%)
Radiotherapy	3 (9.68%)
Anti-vascular treatment	20 (64.52%)
Immunotherapy	2 (6.45%)
Surgery	1 (3.22%)
Third-generation EGFR-TKI therapy	
Before LM	3 (9.68%)
After LM	11 (35.48%)
Before and after LM	12 (38.71%)
None	5 (16.13%)
Combine radiotherapy	
Yes	10 (32.26%)
No	21 (67.74%)
IP number	
1-5	20 (64.52%)
6-10	8 (25.81%)
>10	3 (9.68%)
Combine BEV	
Yes	26 (83.87%)
No	5 (16.13%)

IP, intrathecal chemotherapy of pemetrexed; BEV, bevacizumab.

### 3.2 Clinical manifestations, imaging, and CSF cytology

**Figure 1:**
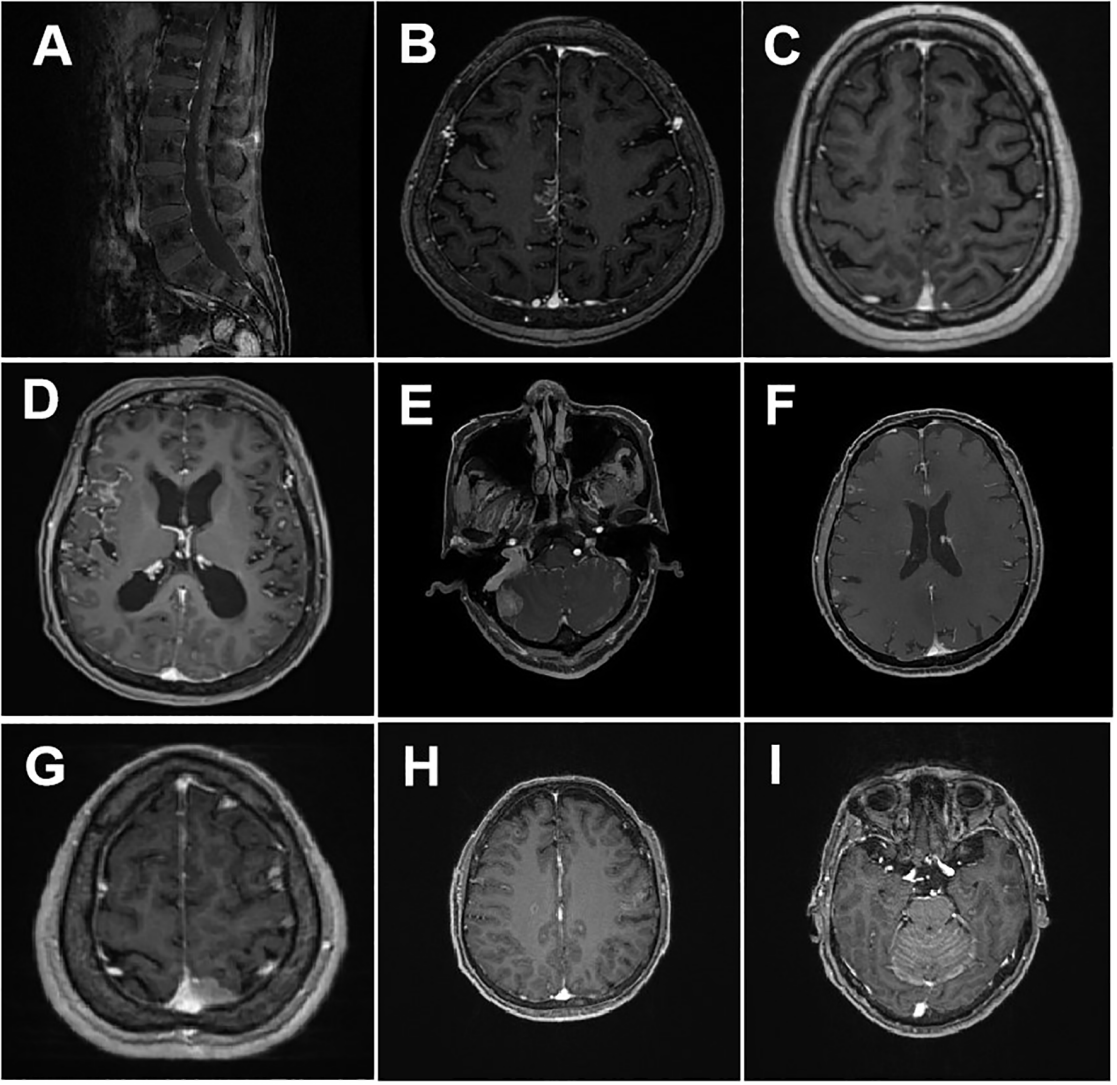
Representative brain MRI of patients with leptomeningeal metastases. **(A)** Meningeal metastasis spreading to the spinal cord, with extensive enhancement of the spinal cones and multiple tubercles in the cauda equina; **(B)** Enhanced MRI showing thickening of the meninges in the right upper frontal midline region, with a slightly decreased T1W signal, a slightly increased T2W signal, and a high T2 flair signal; **(C)** Cranial parenchyma enhancement displaying mild ring-shaped enhancement, with a large patchy high-signal edema band on T2FLAIR surrounding the nodule; **(D)** The meninges of the right frontotemporal lobe showing linear enhancement, along with enlargement of the ventricular system; **(E)** The right cerebellar hemisphere showing a patchy low-signal on T1WI, high-signal on T2WI, low signal on DWI, high signal on T2 FLAIR, and marked enhancement on post-contrast imaging; **(F)** The brain parenchyma and cerebral hemispheric meninges showing uneven thickening and marked enhancement; **(G)** The left parietal dura exhibiting nodular thickening with prominent enhancement; **(H)** Multiple abnormally enhanced nodules were observed in both hemispheres; **(I)** The cerebellar hemispheres displaying multiple abnormally enhanced nodules, with diffuse thickening and enhancement of the cerebellar meninges.

The patients presented with various clinical manifestations, including dizziness and headache in 22 patients, nausea and vomiting in 13, fatigue and difficulty walking in 15, blurred vision and diplopia in five, distortion of the commissure and facial numbness in two, hypophasis in one, hearing loss in two, dysphagia in two, convulsions in four, shoulder and neck pain in one, slow reaction in 10, slurred speech in two, urinary and bowel incontinence in one, upper-limb numbness in two, and increased intracranial pressure in 22. Twenty-six patients (83.87%) exhibited positively enhanced brain MRI, with the majority displaying linear or nodular meningeal enhancement, or accompanied by nodular cerebral parenchymal enhancement, ventricular enlargement, cranial (spinal) nerve enhancement or thickening, enhanced nodules in the spinal arachnoid space, and hydrocephalus (**Figure 1**). Intracranial pressure was increased in 13 patients (41.94%). CSF analysis revealed hypoglycemia in 18 patients (58.06%) and hyperproteinemia in 21 patients(67.74%). Cancer cells were detected in the CSF of all patients.

### 3.3 Treatment

Prior to diagnosis of LM, 13 patients (41.94%) received first - or second-generation EGFR-TKIs, three (9.68%) received third-generation EGFR-TKIs, and 10 (32.26%) received first- to third-generation EGFR-TKIs. One patient (3.2%) received ALK inhibitor (crizotinib), and 11 patients (35.48%) received systemic chemotherapy with or without immunotherapy. After the onset of LM, 23 patients (74.19%) were treated with third-generation EGFR-TKIs (osimertinib, furmonertinib, or aumolertinib), of whom 18 (58.06%) were treated with high-dose third-generation EGFR-TKIs. One patient underwent a ventriculo-peritoneal (VP) shunt surgery. During the entire treatment period, 10 patients (32.26%) received radiotherapy (seven brain stereotactic body radiotherapy (SBRT) before LM, two brain SBRT after LM, one whole brain radiotherapy (WBRT) after LM), and 26 patients (83.87%) were treated with combination of bevacizumab (six before LM, 13 after LM, seven before and after LM) (**Table 2**). Patients received an average of 5.8 intrathecal pemetrexed injections, with 28 patients receiving a dose of 30 mg/injection and three patients receiving a dose of 20 mg/injection. Moreover, 27 patients showed improvement in intracranial symptoms after intrathecal pemetrexed chemotherapy and systemic therapy, with an intracranial control rate of 87.1%; of these, eight (25.81%) had CSF that was either negative for cancer cells or contained only a small amount of residual cellular debris.

**Table 2:** Treatment of patients (N=31).

Patient	Gene mutation	Treatment before LM	Systemic treatment after LM	Number of IP	Response	iPFS(m)	iOS(m)
1	EGFR21 L858R	Gefitinib,Endostar,Osimertinib,BEV+Pemetrexed+Platinum	IP+Anlotinib	4	Improved	5	8
2	EGFR 19Del	Pemetrexed+Platinum,Gefitinib,Osimertinib, BEV,SBRT	IP+Osimertinib	6	Improved	2	2
3	Wild-type (LM was found at initial diagnosis)		IP+Pemetrexed+Platinum	2	Worsened	1	1
4	EGFR21 L858R	Osimertinib	IP+Osimertinib+SBRT	2	Worsened	4	5
5	KRAS+	Pemetrexed+Platinum+BEV,Camrelizumab+Docetaxel+Anlotinib, SBRT	IP+BEV, Anlotinib	6	Improved	6	8
6	EGFR21 L858R	Aumolertinib	IP+Aumolertinib+BEV	3	Improved	7	7
7	EGFR T790M	Gefitinib,Osimertinib,	IP+Osimertinib +BEV	26	Improved	35.2+	35.2+
8	EGFR21 L858R	Osimertinib	IP+Osimertinib	5	Worsened	2	3
9	EGFR 19Del	Aumolertinib,Pemetrexed+Platinum+BEV,Etoposide+Platinum+Anlotinib	IP+Irinotecan+Sintilimab	3	Worsened	2	2
10	EGFR 20ins	Pemetrexed+Platinum+Sintilimab	IP+Furmonertinib+BEV	10	Improved	14.6+	14.6+
11	EGFR21 L858R	Aumolertinib+BEV	IP+Aumolertinib+Icotinib+BEV	4	Improved	14	21+
12	EGFR 20ins	Furmonertinib, Pemetrexed+Platinum+SBRT,TAK788	IP+WBRT,Anlotinib	3	Improved	21	25
13	EGFR20ins	Pemetrexed+Platinum+BEV	IP+BEV+Osimertinib+VP shunt	14	Improved	10	12
14	EGFR 19Del	Gefitinib	IP+Osimertinib+BEV	2	Improved	6	8
15	EGFR21 L858R	Icotinib+BEV,SBRT	IP+Aumolertinib	2	Improved	6	8
16	EGFR21 L858R (LM was found at initial diagnosis)		IP+Osimertinib+BEV	6	Improved	18+	18+
17	EGFR21 L858R (LM was found at initial diagnosis)		IP+Osimertinib+BEV	4	Improved	14	16
18	Wild-type	Pemetrexed+Platinum+Camrelizumab, SBRT+Docetaxel,Anlotinib	IP+Pemetrexed+Platinum	4	Improved	8.7	10
19	EGFR21 L858R (LM was found at initial diagnosis)		IP+Furmonertinib+BEV	8	Improved	20.2+	20.2+
20	EGFR 19Del	Gefitinib, Osimertinib	IP+Osimertinib	5	Improved	8.6	12.2
21	ROSI (LM was found at initial diagnosis)		Crizotinib+BEV, IP+Pemetrexed+Platinum+BEV	3	Improved	25	28
22	EGFR21 L858R	Gefitinib	IP+BEV+OsimertinibPemetrexed+Platinum+Aumolertinib	9	Improved	6.3	11.3
23	EGFR21 L858R	Gefitinib,Furmonertinib,SBRT	IP+Furmonertinib	4	Improved	8.2	8.2
24	EGFR21 L858R	Gefitinib	IP+Osimertinib	6	Improved	5.1	6.1
25	EGFR 19Del	Icotinib,Aumolertinib, SBRT	IP+Aumolertinib+BEV	2	Improved	17	23
26	EGFR 19Del (LM was found at initial diagnosis)		IP+Osimertinib+BEV	3	Improved	9	11
27	EGFR21 L858R	Gefitinib,Osimertinib,SBRT	IP+Osimertinib+BEV	14	Improved	27	29
28	EGFR21 L858R	Gefitinib	IP+Osimertinib+BEV	2	Improved	14.2+	14.2+
29	EGFR 19Del	Gefitinib	IP+Osimertinib+BEV	9	Improved	13	13.6+
30	Wild-type	Docetaxel+Platinum+BEV	IP+Pemetrexed+Platinum+Sintilimab,SBRT	4	Improved	26	28
31	EGFR 20ins	Osimertinib	IP+Osimertinib+BEV	5	Improved	14.2+	14.2+

BEV, bevacizumab; + means the patient is still alive.

### 3.4 Survival and prognosis factors

**Figure 2:**
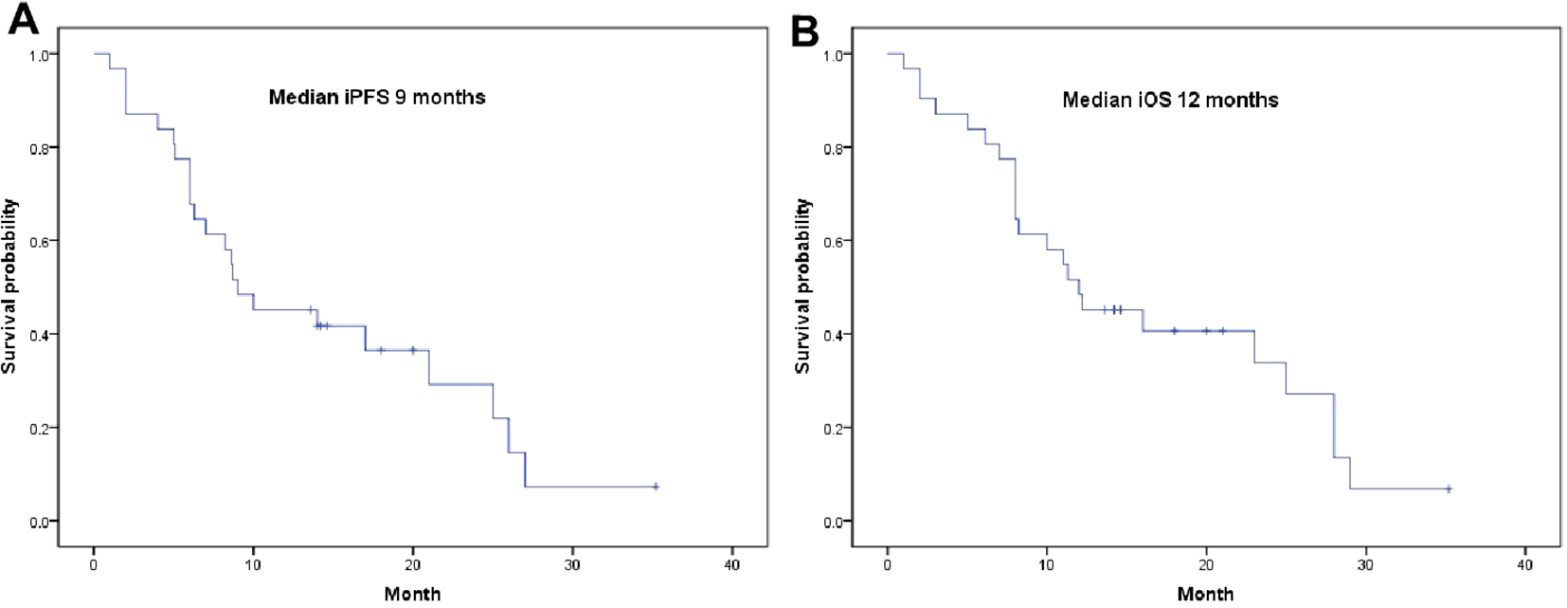
Survival curves for patients. **(A)** Kaplan-Meier curve of intracranial iPFS; **(B)** Kaplan-Meier curve of intracranial iOS.

By the date of the last follow-up, all 31 patients had completed follow-up, with a median follow-up time of 20.4 (1-35) months; 23 patients had died, and eight patients are still alive. The median iPFS was 9 months (95% CI: 2.77-15.23), and the median iOS was 12 months (95% CI: 5.94-18.06 months) (**Figures 2A, B**). The univariate and multivariate analyses showed that combined bevacizumab treatment and ECOG ≤1 were favorable prognostic factors for survival, while sex, age, smoking status, brain metastases, extracerebral metastases, elevated CSF protein levels, gene mutations, positively enhanced brain MRI, and radiotherapy had no significant influence on OS (**Table 3**).

**Table 3:** Prognostic factor analysis of patients (N=31).

Factor	Media iOS (m)	Univariate *P* value	Multivariate *P* value
**Age**		0.756	
<60	11.3		
≥60	12.2		
**Sex**		0.594	
Male	11.3		
Female	12.2		
**ECOG score**		0.001	0.015
0-1	14.6		
≥2	6.1		
**Smoking**		0.223	
Yes	8.7		
No	12		
**CSF pressure**		0.976	
High	11.3		
normal	12.2		
**High protein in CSF**		0.616	
Yes	11		
No	12		
**MRI**		0.653	
Negative	11		
Positive	13.6		
**Brain metastases**		0.660	
Yes	8.2		
No	11.3		
**Extracerebral metastasis**		0.779	
Yes	12		
No	14.6		
**Gene mutation**		0.725	
EGFR	12	Ref	
Negative	10	0.651	
KRAS	8	0.370	
ROS1	28	0.608	
**IP number**		0.215	
1-5	10	Ref	
6-10	11.3	0.538	
>10	29	0.093	
**Combine BEV**		0.001	0.002
Yes	14.2		
No	6.1		
**Combine Radiotherapy**		0.452	
Yes	8.2		
No	12.2		
**Third-generation EGFR-TKI therapy**		0.751	
Yes	12		
No	10		

IP, intrathecal chemotherapy of pemetrexed; BEV, bevacizumab.

### 3.5 Adverse events

Most common AEs were grade 1-2, including leukopenia in 17 (54.84%) patients, nausea in 10 (32.26%), elevated alanine transaminase/aspartate transaminase levels in seven (22.58%), diarrhea in five (16.13%), weakness in eight (25.80%), rash in seven (22.58%), decreased appetite in six (19. 35%), and elevated gamma-GT in five (16.13%); there were three Grade 3 AEs, including two cases of leukopenia, one case of third-degree diarrhea, and one case of third-degree interstitial pneumonia (**Table 4**).

**Table 4:** Adverse events (N= 31).

Adverse event	Any Grade	Grade 1 (n)	Grade 2 (n)	Grade 3 (n)	Grade 4 (n)
leukopenia	17 (54.84%)	11	4	2	0
Nausea	10 (32.26%)	7	3	0	0
Vomiting	6 (19.35%)	4	2	0	0
Elevated ALT/AST	7 (22.58%)	6	1	0	0
Diarrhea	5 (16.13%)	3	1	1	0
Fatigue	8 (25.80%)	6	2	0	0
Rash and acnes	7 (22.58%)	5	2	0	0
Paronychia	2 (6.45%)	2	0	0	0
Stomatitis	3 (9.68%)	2	1	0	0
Decreased appetite	6 (19.35%)	4	2	0	0
Elevated γ-GT	5 (16.13%)	4	1	0	0
Pneumonia	2 (6.45%)	1	0	1	0

ALT, alanine aminotransferase; AST, aspartate aminotransferase; γ-GT, Gamma-glutamyl transpeptidase.

## 4 Discussion

LM is a severe complication of solid tumors, associated with a poor prognosis. The clinical manifestations of LM are complex and vary based on the affected sites (1). Brain parenchymal involvement and meningeal involvement: symptoms include headache, nausea, vomiting, cervical tension, meningeal irritation signs, cognitive impairment, seizures, and limb movement disorders (2). Cerebral neuropathy: symptoms include reduced visual acuity, diplopia, facial numbness, taste and hearing abnormalities, and difficulties with swallowing and articulation (3). Progressive cerebral dysfunction: this may result from increased intracranial pressure and hydrocephalus (4). Urinary and bowel dysfunction: these symptoms arise from spinal membrane invasion (16). In this study, there were 22 cases of cerebral parenchymal and meningeal stimulation, 14 cases of cerebral neuropathy, one case of urinary and bowel incontinence caused by meningeal invasion, and 22 cases of intracranial hypertension. Early diagnosis of LM is difficult because of the lack of specificity of clinical manifestations; consequently, LM is prone to misdiagnosis or missed diagnosis. Cranial MRI is essential for the diagnosis of LM, especially enhanced MRI, which has a specificity of 77% and a sensitivity of 76% in patients with LM harboring solid tumors (17). Typical cranial MRI enhancement may show enhancement of the soft meninges and ventricular meninges; plaques, nodules, or masses in the subarachnoid or intraventricular spaces; enhancement or thickening of the cranial (spinal) nerves; ventricular dilatation; and hydrocephalus (18, 19). Owing to the enhanced contrast of pia meningeal MRI caused by external stimulation, MRI is recommended before performing a lumbar puncture. CSF cytology is the gold standard for the diagnosis of LM, but its sensitivity is lower than that of MRI, with malignant cells detected in only 50-67% of patients. Nevertheless, the sensitivity can be increased to 80-90% after 2-3 consecutive CSF examinations (20, 21); 90% of patients with LM exhibit abnormal levels of CSF cells and protein expression (22). In this study, cancer cells were detected in the initial lumbar puncture of all 31 patients, and elevated levels of CSF protein were observed in 21 patients (67.74%). Cell-free DNA is an emerging diagnostic technique with higher sensitivity than CSF cytology and MRI (23, 24), providing valuable genetic information. This is very important for early diagnosis, treatment guidance, and the evaluation of therapeutic efficacy and tumor burden.

Treatment of LM aims to improve neurological symptoms and prolong OS, taking into account the patient’s histology, molecular typing, clinical presentation, MRI, neurological function, and prognosis. Radiotherapy is the primary treatment for LM, including WBRT and SBRT. WBRT is commonly used in patients with extensive nodal or linear meningeal metastases and is considered a palliative treatment for symptomatic relief. However, WBRT may not provide a significant survival benefit and can lead to cognitive decline (25, 26). SBRT may be considered for focal symptomatic disorders, such as cauda equina syndrome and cranial neuropathy (27). Our study also showed that radiotherapy had no significant influence on OS. VP shunt is an effective treatment for hydrocephalus and intracranial hypertension. A study of 31 patients with leptomeningeal metastasis-related hydrocephalus showed that VP shunt rapidly improved symptoms in 90.3% of patients, with a median OS of 7.7 months after the onset of LM (28). Another study with larger data (70 patients) found that VP shunt resulted in symptomatic improvement in 50% of patients, with complete resolution of symptoms in 34% of patients; however, VP shunt had many adverse effects, including infection in eight patients, shunt malfunction in eight patients, and the need for shunt repair in 17 patients, with a median OS after VP of 4.1 months (29). In this study, one patient underwent a VP shunt owing to refractory intracranial hypertension and experienced rapid improvement of craniocerebral symptoms; however, this patient subsequently developed malignant pleural and abdominal effusions leading to death after 1 month. Systemic chemotherapy combined with antivascular or immunotherapeutic agents is the primary treatment option for NSCLC patients with negative driver gene mutation and LM. However, the presence of the BBB hinders most chemotherapeutic agents from penetrating the pia mater, thereby limiting their therapeutic efficacy; therefore, a combination therapy approach is required. In this study, there were three patients with negative driver gene mutation, of which one received intrathecal pemetrexed injection in conjunction with systemic chemotherapy, immunotherapy, and SBRT. This comprehensive treatment strategy resulted in an impressive iPFS of 26 months.

Compared with conventional chemotherapeutic agents, third-generation EGFR-TKIs exhibit superior CSF permeability and intracranial response rates. In patients with EGFRm NSCLC and LM, osimertinib shows superior efficacy compared to first- and second-generation EGFR-TKIs, significantly improving PFS and OS (30, 31), regardless of the presence of T790M mutations in the CSF. A retrospective study involving 304 patients with EGFR-mutated NSCLC showed that among the 116 patients receiving osimertinib and the 188 patients receiving first- or second-generation EGFR-TKIs, osimertinib treatment reduced the incidence of LM by 67%,and osimertinib treatment was an independent significant indicator of reduced LM incidence (8). Aumolertinib has high BBB penetration owing to the structural introduction of cyclopropyl; in a mouse model of EGFRm NSCLC brain metastases, aumolertinib exposure in the brain was more than seven times higher than plasma exposure (32). In the phase II APOLLO study, analysis of measurable lesions in brain metastases suggested that the central nervous system (CNS) objective remission rate (ORR) and CNS disease control rates were 60.9% (95% CI: 38.5-80.3) and 91.3% (95% CI: 72.0-98. 9), respectively (33). In the AENEAS CNS full analysis set, the mPFS for patients treated with aumolertinib and gefitinib in the first-line was 29 months and 8.3 months, respectively (34). Furmonertinib is an irreversible third-generation EGFR-TKI whose metabolites enter the brain and persist in brain tissue for a long period (35). A prospective real-world study of furmonertinib in patients with LM from EGFRm NSCLC found a median OS of 8.43 months (95% CI: 5.48-11.39 months) following treatment with furmonertinib, with an LM objective response rate of 50.0% and a disease control rate of 92.1%, respectively (36). In this study, three patients received third-generation EGFR-TKIs prior to LM; 11, after LM; 12, both before and after LM; and five, did not receive third-generation EGFR-TKIs. The median OS for the groups using third-generation EGFR-TKIs before, after, and before and after LM, as well as for those not using any third-generation EGFR-TKIs, was 9, 14, 12, and 10 months, respectively. The relatively short OS with third-generation EGFR-TKIs before LM, which is inconsistent with previous studies, may be related to the small sample size.

EGFR-TKIs also encounter the challenge of drug resistance, with 40% of relapses occurring after treatment with first- and/or second-generation targeted agents (37). This resistance is mainly due to the inability of standard doses of the drug to achieve effective CSF concentrations. Therefore, high-dose EGFR-TKIs have become a viable therapeutic option for patients with NSCLC and LM after failure of standard-dose EGFR-TKI treatment (38, 39). The study found that administering 160 mg of osimertinib to patients with EGFRm NSCLC and LM who had progressed after prior EGFR-TKI therapy resulted in a remission duration of 8.3 months, an ORR of 41%, a median PFS of 8.6 months, and a median OS of 11.0 months, with a manageable safety profile (40). EGFR-TKIs combined with anti-vascular drug therapy shown to improve treatment response. Professor Jiang concluded that osimertinib in combination with bevacizumab in patients with NSCLC and LM also showed sustained clinical and radiological responses at 10 months. (41). However, combination immunotherapy with EGFR-TKIs is ineffective in patients with NSCLC and EGFR-sensitive mutations, increasing the risk of treatment (42). In this study, 23 patients received third-generation EGFR-TKI therapy after LM, of whom 18 received high-dose third-generation EGFR-TKI therapy, and drug resistance was observed in 19. Further second-generation gene sequencing of lung tumors or CSF revealed a RET gene fusion in one patient, MET amplification in one patient, EGFR20 C797s mutation in one patient, TP53 mutation in three patients, EGFR amplification in one patient, and small cell transformation in one patient. One patient retained the original mutation, while the remaining individuals refused further genetic sequencing.

After patients developed LM, intrathecal pemetrexed injections were administered in combination with high-dose third-generation EGFR-TKIs, a RET inhibitor, a MET inhibitor, bevacizumab, first- and third-generation EGFR-TKIs, or intravenous chemotherapy. There is no consensus on the optimal administration frequency and concentration of intrathecal pemetrexed injection, and previous studies have primarily used 10-50 mg per administration (13, 14). Considering the necessity for patients to undergo combination therapies, pemetrexed was administered at a dosage of 20-30 mg/dose in the patients of this study. Intrathecal pemetrexed injection chemotherapy was administered 2-4 times in the first month, and 1-2 times every month thereafter. After intrathecal pemetrexed chemotherapy, 27 patients experienced significant relief from intracranial symptoms, and eight patients had cancer cells disappeared in their CSF. As a result, some patients refused intrathecal injections after symptom relief, whereas others opted for intermittent intrathecal injections because of recurrent cranial symptoms. The mean number of intrathecal injections in patients was 5.8, with a median iPFS of 9 months (95% CI:2.77-15.23) and a median iOS of 11 months (95% CI:5.94-18.06 months),which was better than the previously reported OS of 3-8.8 months (43, 44).

EGFR-TKI use is a significant prognostic indicator of good survival, while poor physical status, elevated CSF protein levels, and elevated CSF leukocyte counts suggest poor outcomes (45). In an analysis of 155 patients with LM, advanced age (>60 years) and elevated CSF albumin levels were identified as treatment-independent predictors of poor survival (46). In our study, univariate and multivariate analyses showed that the combination of bevacizumab was associated with a good survival prognosis, while ECOG ≥ 2 was a significant predictor of poor survival. As the number of intrathecal pemetrexed injections increased, the median iOS was prolonged, but there was no statistical difference. In terms of safety, most of the manifestations were grade 1-2 AEs, including nausea, vomiting, fatigue, rash and acnes, paronychia, elevated ALT/AST, and there were four cases (12.9%) of grade 3 AEs (including two cases of leukopenia, one case of diarrhea, and one case of interstitial pneumonitis), which were mainly related to high doses of the targeted drug. However, as a single-center, retrospective study with a small sample size, it had some shortcomings. In addition, the dose and frequency of pemetrexed administration were inconsistent.

In conclusion, the combination of intrathecal pemetrexed chemotherapy with systemic therapy represents a promising strategy with manageable safety for the treatment of LM in patients with NSCLC.

## References

[1] Olson ME Chernik NL Posner JB. Infiltration of the leptomeninges by systemic cancer. A clinical and pathologic study. Arch Neurol. (1974) 30:122–37. doi: 10.1001/archneur.1974.00490320010002 4405841

[2] Cheng H Perez-Soler R. Leptomeningeal metastases in non-small-cell lung cancer. Lancet Oncol. (2018) 19:e43–55. doi: 10.1016/S1470-2045(17)30689-7 29304362

[3] Seong M Park S Kim ST Park SG Kim YK Kim HJ. Diagnostic accuracy of MR imaging of patients with leptomeningeal seeding from lung adenocarcinoma based on 2017 RANO proposal: added value of contrast-enhanced 2D axial T2 FLAIR. J neuro-oncol. (2020) 149:367–72. doi: 10.1007/s11060-020-03617-2 32897466

[4] Reckamp KL. Targeted therapy for patients with metastatic non-small cell lung cancer. J Natl Compr Cancer Network: JNCCN. (2018) 16:601–4. doi: 10.6004/jnccn.2018.0046 29784736

[5] Li YS Jiang BY Yang JJ Tu HY Zhou Q Guo WB. Leptomeningeal metastases in patients with NSCLC with EGFR mutations. J Thorac Oncol. (2016) 11:1962–9. doi: 10.1016/j.jtho.2016.06.029 27539328

[6] Morris PG Reiner AS Szenberg OR Clarke JL Panageas KS Perez HR. Leptomeningeal metastasis from non-small cell lung cancer: survival and the impact of whole brain radiotherapy. J Thorac Oncol. (2012) 7:382–5. doi: 10.1097/JTO.0b013e3182398e4f 22089116

[7] Yang JT Wijetunga NA Pentsova E Wolden S Young RJ Correa D. Randomized phase II trial of proton craniospinal irradiation versus photon involved-field radiotherapy for patients with solid tumor leptomeningeal metastasis. J Clin Oncol. (2022) 40:3858–67. doi: 10.1200/JCO.22.01148 PMC9671756 35802849

[8] Wang X Cai J Zeng Z Liu A. Efficacy of osimertinib for preventing leptomeningeal metastasis derived from advanced EGFR-mutated non-small cell lung cancer: a propensity-matched retrospective study. BMC Cancer. (2021) 21:873. doi: 10.1186/s12885-021-08581-2 34330231 PMC8325312

[9] Shetty V Babu S. Management of CNS metastases in patients with EGFR mutation-positive NSCLC. Indian J Cancer. (2019) 56:S31–s37. doi: 10.4103/ijc.IJC_455_19 31793440

[10] Rollins KD Lindley C. Pemetrexed: a multitargeted antifolate. Clin Ther. (2005) 27:1343–82. doi: 10.1016/j.clinthera.2005.09.010 16291410

[11] Choi M Keam B Ock CY Kim M Kim TM Kim DW. Pemetrexed in the treatment of leptomeningeal metastasis in patients with EGFR-mutant lung cancer. Clin Lung Cancer. (2019) 20:e442–51. doi: 10.1016/j.cllc.2019.03.005 31010639

[12] Sun JM Nam MH Chung JY Im B Lee SY Suh YL. Safety and pharmacokinetics of intrathecal administration of pemetrexed in rats. Cancer chemother Pharmacol. (2011) 68:531–8. doi: 10.1007/s00280-010-1522-7 21107572

[13] Pan Z Yang G Cui J Li W Li Y Gao P. A pilot phase 1 study of intrathecal pemetrexed for refractory leptomeningeal metastases from non-small-cell lung cancer. Front Oncol. (2019) 9:838. doi: 10.3389/fonc.2019.00838 31544065 PMC6730526

[14] Fan C Zhao Q Li L Shen W Du Y Teng C. Efficacy and safety of intrathecal pemetrexed combined with dexamethasone for treating tyrosine kinase inhibitor-failed leptomeningeal metastases from EGFR-mutant NSCLC-a prospective, open-label, single-arm phase 1/2 clinical trial (Unique identifier: chiCTR1800016615). J Thorac Oncol. (2021) 16:1359–68. doi: 10.1016/j.jtho.2021.04.018 33989780

[15] Chamberlain M Junck L Brandsma D Soffietti R Rudà R Raizer J. Leptomeningeal metastases: a RANO proposal for response criteria. Neuro-oncology. (2017) 19:484–92. doi: 10.1093/neuonc/now183 PMC5464328 28039364

[16] Shi Y Sun Y Yu J Ding C Ma Z Wang Z. China experts consensus on the diagnosis and treatment of brain metastases of lung cancer (2017 version). Zhongguo fei ai za zhi = Chin J Lung Cancer. (2017) 20:1–13. doi: 10.3779/j.issn.1009-3419.2017.01.01 PMC5973287 28103967

[17] Straathof CS de Bruin HG Dippel DW Vecht CJ. The diagnostic accuracy of magnetic resonance imaging and cerebrospinal fluid cytology in leptomeningeal metastasis. J Neurol. (1999) 246:810–4. doi: 10.1007/s004150050459 10525979

[18] Chou MS Tsai TC Lin MB Liu GC Howng SL. MRI manifestations of leptomeningeal metastasis. Gaoxiong yi xue ke xue za zhi = Kaohsiung J Med Sci. (1994) 10:186–93. 8007048

[19] Le Rhun E Weller M Brandsma D Van den Bent M de Azambuja E Henriksson R. EANO-ESMO Clinical Practice Guidelines for diagnosis, treatment and follow-up of patients with leptomeningeal metastasis from solid tumors. Ann Oncol. (2017) 28:iv84–99. doi: 10.1093/annonc/mdx221 28881917

[20] Wasserstrom WR Glass JP Posner JB. Diagnosis and treatment of leptomeningeal metastases from solid tumors: experience with 90 patients. Cancer. (1982) 49:759–72. doi: 10.1002/1097-0142(19820215)49:4<759::AID-CNCR2820490427>3.0.CO;2-7 6895713

[21] Le Rhun E Massin F Tu Q Bonneterre J Bittencourt Mde C Faure GC. Development of a new method for identification and quantification in cerebrospinal fluid of Malignant cells from breast carcinoma leptomeningeal metastasis. BMC Clin Pathol. (2012) 12:21. doi: 10.1186/1472-6890-12-21 23145812 PMC3539901

[22] Brandsma D Voest EE de Jager W Bonfrer H Algra A Boogerd W. CSF protein profiling using Multiplex Immuno-assay: A potential new diagnostic tool for leptomeningeal metastases. J Neurol. (2006) 253:1177–84. doi: 10.1007/s00415-006-0187-y 16998648

[23] Jiang BY Li YS Guo WB Zhang XC Chen ZH Su J. Detection of driver and resistance mutations in leptomeningeal metastases of NSCLC by next-generation sequencing of cerebrospinal fluid circulating tumor cells. Clin Cancer Res. (2017) 23:5480–8. doi: 10.1158/1078-0432.CCR-17-0047 28606923

[24] Zhao Y He JY Zou YL Guo XS Cui JZ Guo L. Evaluating the cerebrospinal fluid ctDNA detection by next-generation sequencing in the diagnosis of meningeal Carcinomatosis. BMC Neurol. (2019) 19:331. doi: 10.1186/s12883-019-1554-5 31856745 PMC6924020

[25] Yan W Liu Y Li J Han A Kong L Yu J. Whole brain radiation therapy does not improve the overall survival of EGFR-mutant NSCLC patients with leptomeningeal metastasis. Radiat Oncol (London England). (2019) 14:168. doi: 10.1186/s13014-019-1376-z PMC6744654 31521171

[26] Zhen J Wen L Lai M Zhou Z Shan C Li S. Whole brain radiotherapy (WBRT) for leptomeningeal metastasis from NSCLC in the era of targeted therapy: a retrospective study. Radiat Oncol (London England). (2020) 15:185. doi: 10.1186/s13014-020-01627-y PMC7393872 32736566

[27] Horbinski C Nabors LB Portnow J Baehring J Bhatia A Bloch O. NCCN guidelines^®^ Insights: central nervous system cancers, version 2.2022. J Natl Compr Cancer Network: JNCCN. (2023) 21:12–20. doi: 10.6004/jnccn.2023.0002 36634606

[28] Mitsuya K Nakasu Y Hayashi N Deguchi S Takahashi T Murakami H. Palliative cerebrospinal fluid shunting for leptomeningeal metastasis-related hydrocephalus in patients with lung adenocarcinoma: A single-center retrospective study. PloS One. (2019) 14:e0210074. doi: 10.1371/journal.pone.0210074 30629621 PMC6328154

[29] Kim HS Park JB Gwak HS Kwon JW Shin SH Yoo H. Clinical outcome of cerebrospinal fluid shunts in patients with leptomeningeal carcinomatosis. World J Surg Oncol. (2019) 17:59. doi: 10.1186/s12957-019-1595-7 30917830 PMC6438037

[30] Zhang M Ma W Liu H Jiang Y Qin L Li W. Osimertinib improves overall survival in patients with leptomeningeal metastases associated with EGFR-mutated non-small-cell lung cancer regardless of cerebrospinal fluid T790M mutational status. Evidence-Based complementary Altern medicine: eCAM. (2021) 2021:6968194. doi: 10.1155/2021/6968194 PMC8397557 34457028

[31] Lee J Choi Y Han J Park S Jung HA Su JM. Osimertinib improves overall survival in patients with EGFR-mutated NSCLC with leptomeningeal metastases regardless of T790M mutational status. J Thorac Oncol. (2020) 15:1758–66. doi: 10.1016/j.jtho.2020.06.018 32652216

[32] Zhang Y Zhang Y Niu W Ge X Huang F Pang J. Experimental study of almonertinib crossing the blood-brain barrier in EGFR-mutant NSCLC brain metastasis and spinal cord metastasis models. Front Pharmacol. (2021) 12:750031. doi: 10.3389/fphar.2021.750031 34630120 PMC8497791

[33] Lu S Wang Q Zhang G Dong X Yang CT Song Y. Efficacy of aumolertinib (HS-10296) in patients with advanced EGFR T790M+ NSCLC: updated post-national medical products administration approval results from the APOLLO registrational trial. J Thorac Oncol. (2022) 17:411–22. doi: 10.1016/j.jtho.2021.10.024 34801749

[34] Lu S Dong X Jian H Chen J Chen G Sun Y. Central nervous system efficacy of aumolertinib versus gefitinib in patients with untreated, EGFR-mutated, advanced non-small cell lung cancer: data from a randomized phase III trial (AENEAS). Cancer Commun (London England). (2024) 44:1005–17. doi: 10.1002/cac2.12594 PMC11492357 39016053

[35] Shi Y Chen G Wang X Liu Y Wu L Hao Y. Furmonertinib (AST2818) versus gefitinib as first-line therapy for Chinese patients with locally advanced or metastatic EGFR mutation-positive non-small-cell lung cancer (FURLONG): a multicenter, double-blind, randomized phase 3 study. Lancet Respir Med. (2022) 10:1019–28. doi: 10.1016/S2213-2600(22)00168-0 35662408

[36] Chen H Yang S Wang L Wu Y Wu Y Ma S. High-dose furmonertinib in patients with EGFR-mutated NSCLC and leptomeningeal metastases: A prospective real-world study. J Thorac Oncol. (2025) 20:65–75. doi: 10.1016/j.jtho.2024.09.1385 39260521

[37] Pellerino A Brastianos PK Rudà R Soffietti R. Leptomeningeal metastases from solid tumors: recent advances in diagnosis and molecular approaches. Cancers. (2021) 13. doi: 10.3390/cancers13122888 PMC8227730 34207653

[38] Kawamura T Hata A Takeshita J Fujita S Hayashi M Tomii K. High-dose erlotinib for refractory leptomeningeal metastases after failure of standard-dose EGFR-TKIs. Cancer chemother Pharmacol. (2015) 75:1261–6. doi: 10.1007/s00280-015-2759-y 25921002

[39] Wu H Zhang Q Zhai W Chen Y Yang Y Xie M. Effectiveness of high-dose third-generation EGFR-tyrosine kinase inhibitors in treating EGFR-mutated non-small cell lung cancer patients with leptomeningeal metastasis. Lung Cancer (Amsterdam Netherlands). (2024) 188:107475. doi: 10.1016/j.lungcan.2024.107475 38266613

[40] Yang JCH Kim SW Kim DW Lee JS Cho BC Ahn JS. Osimertinib in patients with epidermal growth factor receptor mutation-positive non-small-cell lung cancer and leptomeningeal metastases: the BLOOM study. J Clin Oncol. (2020) 38:538–47. doi: 10.1200/JCO.19.00457 PMC7030895 31809241

[41] Jiang T Xu X Chen X Ding N Hu Q Zhou C. Osimertinib in combination with bevacizumab in EGFR-Mutated NSCLC with leptomeningeal metastases. Trans Lung Cancer Res. (2020) 9:2514–7. doi: 10.21037/tlcr-20-984 PMC7815344 33489813

[42] Yang JC Shepherd FA Kim DW Lee GW Lee JS Chang GC. Osimertinib Plus Durvalumab versus Osimertinib Monotherapy in EGFR T790M-Positive NSCLC following Previous EGFR TKI Therapy: CAURAL Brief Report. J Thorac Oncol. (2019) 14:933–9. doi: 10.1016/j.jtho.2019.02.001 30763730

[43] Gwak HS Joo J Kim S Yoo H Shin SH Han JY. Analysis of treatment outcomes of intraventricular chemotherapy in 105 patients for leptomeningeal carcinomatosis from non-small-cell lung cancer. J Thorac Oncol. (2013) 8:599–605. doi: 10.1097/JTO.0b013e318287c943 23422833

[44] Nakagawa H Fujita T Kubo S Izumoto S Nakajima Y Tsuruzono K. Ventriculolumbar perfusion chemotherapy with methotrexate and cytosine arabinoside for meningeal carcinomatosis: a pilot study in 13 patients. Surg Neurol. (1996) 45:256–64. doi: 10.1016/0090-3019(95)00403-3 8638223

[45] Lee SJ Lee JI Nam DH Ahn YC Han JH Sun JM. Leptomeningeal carcinomatosis in non-small-cell lung cancer patients: impact on survival and correlated prognostic factors. J Thorac Oncol. (2013) 8:185–91. doi: 10.1097/JTO.0b013e3182773f21 23328548

[46] Herrlinger U Förschler H Küker W Meyermann R Bamberg M Dichgans J. Leptomeningeal metastasis: survival and prognostic factors in 155 patients. J neurological Sci. (2004) 223:167–78. doi: 10.1016/j.jns.2004.05.008 15337619

